# Risk of dementia in patients with end-stage renal disease under maintenance dialysis—a nationwide population-based study with consideration of competing risk of mortality

**DOI:** 10.1186/s13195-019-0486-z

**Published:** 2019-04-09

**Authors:** Yi-Ting Kuo, Chung-Yi Li, Junne-Ming Sung, Chiung-Chih Chang, Jung-Der Wang, Chien-Yao Sun, Jia-Ling Wu, Yu-Tzu Chang

**Affiliations:** 1 Department of Internal Medicine, National Cheng Kung University Hospital, College of Medicine, National Cheng Kung University, Tainan, Taiwan; 2 Department of Public Health, National Cheng Kung University, College of Medicine, Tainan, Taiwan; 3 Department of Public Health, College of Public Health, China Medical University, Taichung, Taiwan; 4 Department of Internal Medicine, College of Medicine, National Cheng Kung University, Tainan, Taiwan; 5 Department of Neurology, Cognition and Aging Center, Kaohsiung Chang Gung Memorial Hospital, Chang Gung University College of Medicine, Kaohsiung, Taiwan; 6 Department of Environmental and Occupational Health, National Cheng Kung University Hospital, Tainan, Taiwan

**Keywords:** Dementia, End-stage renal disease, Competing risk analysis, Alzheimer’s disease, Vascular dementia

## Abstract

**Background:**

Dementia is prevalent in the end-stage renal disease (ESRD) population. However, it is still not clarified whether ESRD is one of the etiology of dementia or its attributable effect on the cumulative risk of dementia. Meanwhile, the effect of competing risk of mortality should be taken into consideration when performing epidemiologic analyses among populations with high risk of mortality.

**Methods:**

By using the National Health Insurance Research Database (1998–2010), we identified 927,142 non-ESRD individuals and 99,158 ESRD patients to investigate the effect of ESRD on the risk of dementia. Age- and sex-specific incidence rates (IRs) and cumulative incidence rates (CIRs) were first compared between these two cohorts. Competing risk analyses including cause-specific and subdistribution proportional hazards models were then constructed with adjustments for potential confounders.

**Results:**

The overall IR and CIR of dementia were much higher in the ESRD group than in the non-ESRD group (10.73 vs. 1.40 per 1000 person-years and 0.061 vs. 0.017, respectively, both *P* < 0.0001). Results from the multivariable cause-specific hazard models suggested that ESRD was one of the etiological factors for dementia (cause-specific hazard ratio [csHR] : 2.06 [95% CI : 1.95–2.17]). However, the subdistribution HR (sdHR) of ESRD was 0.51 (95% Cl : 0.49–0.54), which indicated the lower cumulative incidence risk of dementia in ESRD patients. The inverse relationship between csHR and sdHR could be explained by the high mortality rate in the ESRD population. These findings were also essentially consistent across various subgroup analyses according to selected confounders, as well as in the analyses that limited dementia diagnoses made by neurologists or psychologists.

**Conclusions:**

Although ESRD appears directly associated with the risk of dementia, the high competing mortality means that primary prevention of comorbidity associated with dementia may be more effective in reducing overall dementia in the general population, which may also potentially reduce the incidence of ESRD and prevent death from multimorbidity when affected by ESRD.

**Electronic supplementary material:**

The online version of this article (10.1186/s13195-019-0486-z) contains supplementary material, which is available to authorized users.

## Introduction

Patients with end-stage renal disease (ESRD) have higher prevalence rates of cognitive impairment and dementia than the general population [1–4]. Since the brain and kidney have similar microvascular structures and hemodynamic fluctuations, both organs share some common risk factors for vascular damage, including inflammation, atherogenesis, and oxidative stress [5–7]. It might explain why patients with ESRD are also inclined to develop a wide range of diverse neurological disorders, including cognitive impairment and dementia [8, 9]. Indeed, the prevalence of cognitive impairment could be as high as 87% in the ESRD population [10]. Timely identification of dementia in ESRD patients is important, because dementia is associated with many adverse outcomes, including disability, hospitalization, impaired quality of life, dialysis withdrawal, and mortality [1, 11–15]. Given the increased life expectancy and aging of the population worldwide, the burden of dementia in the ESRD population is expected to increase, especially in Asia where the incidence rate of dementia is higher than other geographic areas [16].

To address the considerable medical expenditure and social burden resulting from the lack of an effective cure for dementia, prevention strategies are needed to identify the factors associated with dementia and provide alternative treatment approaches. Numerous factors are considered to increase the risk of dementia in the general population [17–19], and these are also prevalent in the ESRD population. Therefore, the relationship between the risk of dementia and ESRD might be confounded by these common risk factors in the ESRD population. While many previous studies assessed the prevalence of dementia in the ESRD population, very few studies were carried out to investigate the incidence of dementia [20, 21]. Moreover, even the studies investigating the association between non-dialysis-dependent chronic kidney disease (CKD-ND) and risk of cognitive decline revealed conflicting results [22]. To clarify whether ESRD is one of the underlying etiology of dementia and to quantify the cumulative risk of dementia in the ESRD population might facilitate a more appropriate allocation of healthcare expenditure for providing timely prevention and therapeutic strategies and thus improve clinical outcomes of ESRD patients. In this study, we aimed to assess the attributable effect of ESRD on the etiology and cumulative incidence of dementia by using two nationally representative cohorts and competing risk analytical methods.

## Methods

### Data source

The data in this study originated from the National Health Insurance (NHI) Research Database in Taiwan. The NHI is a nationwide healthcare program, which was instituted in 1995 and covered 99.9% of the residents as of 2014 [23]. Nearly all kinds of medical services, including outpatient and inpatient services, medications, and intervention procedures, are reimbursed by the NHI. The claims’ information related to each beneficiary is recorded in detail and then maintained in the NHI Research Database after combining with the individuals’ demographic profiles (birth date, sex, place of residence). To guarantee privacy rights, the identifying information for each beneficiary is encrypted before releasing it to researchers. The diagnoses in this database are defined based on the codes of the *International Classification of Disease, Ninth Edition* (ICD-9). Several studies have validated the accuracy of the NHI Research Database and shown how this data has contributed to numerous high-quality studies [24–28]. This study was conducted after approval by the Institutional Review Board of the National Cheng Kung University Hospital (A-ER-101-089).

### Identification of the study population

**Fig. 1:**
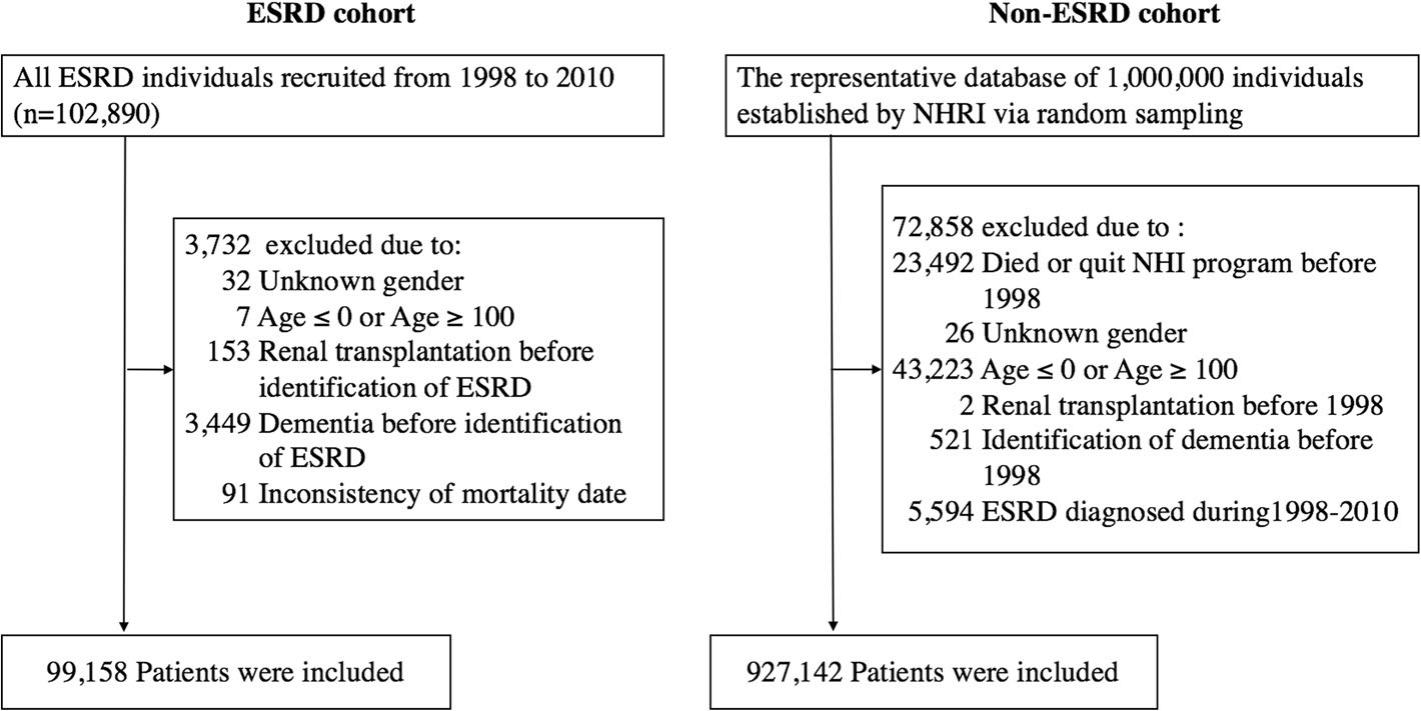
Flow chart of the establishment of end-stage renal disease (ESRD) and non-ESRD populations. NHRI, National Health Research Institute; NHI, National Health Insurance

The most straightforward study design is to compare the difference in risk of dementia between matched pairs generated from the ESRD and non-ESRD populations by various matching statistical techniques. However, the matching approach can lead to limited sample size being enrolled into the final analysis if the distribution of potential confounders is quite different between the comparators (as in our study, shown in Table 1 and Additional file 1: Table S1). In addition, the enrollment of small sample size into the analysis will also limit the generalization of the study results to the whole population. Therefore, we chose to directly compare the risk of dementia between ESRD and non-ESRD populations without any matching process as an alternative design for analysis. Two databases were used for the analysis. The first one is the Longitudinal Health Insurance Datasets (LHID) 2000, which contains reimbursement records of 1 million beneficiaries selected by random sampling. It acts as a representative cohort for more than 23.75 million people with insurance during 1996–2000. The second database contains a specific cohort of all ESRD patients registered in the Catastrophic Illness Datasets and receiving more than three consecutive months of dialysis therapy during January 1, 1998, to December 31, 2010. Any ESRD patient will be certificated for catastrophic illness if he or she is regarded as in irreversible status. This process is reviewed by expert nephrologists according to the relevant clinical information, including underlying cause of kidney damage, indication of initiating dialysis, and laboratory and sonographic findings, to determine the need for long-term dialysis before approval. Any individual in the LHID 2000 was excluded from the data used in this study if he or she had missing or extreme values of age or gender, died or quit NHI before 1998, was diagnosed as having ESRD during 1998–2010, and received renal transplantation (ICD-9: V42.0) or a diagnosis of dementia (ICD-9: 290.0-290.4, 294.0, 294.1, 294.9, and 331.0-331.2) before 1998 (Fig. 1). In the ESRD population, patients were excluded if they had missing or extreme values of age or gender, received renal transplantation, or had a diagnosis of dementia before the identification of ESRD, or inconsistent mortality dates (Fig. 1). The index date of enrollment was the first date of commencing dialysis for three consecutive months in the ESRD population, and January 1, 1998, in the non-ESRD population. In addition, we did not confine our study population to those aged older than 65 years because some individuals were still at risk for dementia as early as in their 20–30 years of age [29].

**Table 1:** Comparison of baseline demographics and selected comorbidities between end-stage renal disease (ESRD) and non-ESRD populations

	Non-ESRD population	ESRD population	*P* value
Number of patients	927,142	99,158	–
Age, no. (%)			
Mean (SD)	32.24 (19.67)	61.55 (14.20)	< 0.0001
≤ 18 years	242,459 (26.15)	264 (0.27)	< 0.0001
18–29 years	208,892 (22.53)	1937 (1.95)	
30–39 years	168,804 (18.21)	4803 (4.84)	
40–49 years	132,851 (14.33)	12,779 (12.89)	
50–59 years	70,873 (7.64)	21,698 (21.88)	
60–69 years	57,358 (6.19)	25,477 (25.69)	
70–79 years	35,248 (3.80)	23,324 (23.52)	
≥ 80 years	10,657 (1.15)	8876 (8.95)	
Sex, no. of male (%)	475,274 (51.26)	49,275 (49.69)	< 0.0001
Median (IQR) length of follow-up (years)	13.00 (0.00)	2.87 (4.22)	< 0.0001
Comorbidities, no. (%)			
Diabetes mellitus	16,792 (1.81)	51,995 (52.44)	< 0.0001
Stroke	3143 (0.34)	10,658 (10.75)	< 0.0001
Anemia	3936 (0.42)	52,573 (53.02)	< 0.0001
Heart failure	2169 (0.23)	28,675 (28.92)	< 0.0001
Hypertension	35,026 (3.78)	77,084 (77.74)	< 0.0001
Hyperlipidemia	4299 (0.46)	26,841 (27.07)	< 0.0001
Coronary artery disease	4444 (0.48)	24,147 (24.35)	< 0.0001
Peripheral vascular disease	400 (0.04)	5113 (5.16)	< 0.0001
Malignancy	3904 (0.42)	8375 (8.45)	< 0.0001
Depression	517 (0.06)	3038 (3.06)	< 0.0001
Obstructive sleep apnea	65 (0.01)	265 (0.27)	< 0.0001
Insomnia	449 (0.05)	11,813 (11.91)	< 0.0001
Alcoholism	1144 (0.12)	1058 (1.07)	< 0.0001
Traumatic brain injury	7830 (0.84)	1504 (1.52)	< 0.0001
Parkinson’s disease	396 (0.04)	1189 (1.20)	< 0.0001
Myocardial infarction	769 (0.08)	4242 (4.28)	< 0.0001
Atrial fibrillation	943 (0.10)	3310 (3.34)	< 0.0001
Hyperthyroidism	459 (0.05)	796 (0.80)	< 0.0001
Hypothyroidism	107 (0.01)	1021 (1.03)	< 0.0001

*SD* standard deviation, *IQR* interquartile range

### Measurement of the outcome variable

The outcome of interest was newly diagnosed dementia during the follow-up period. Individuals were defined as having dementia if the diagnosis was recorded once or more at inpatient care or twice or more at ambulatory care with a minimum interval of > 30 days within 1 year. The study period of both ESRD and non-ESRD populations was from January 1, 1998, to December 31, 2010. The date of the end of study or withdrawal from the NHI program not due to mortality was treated as censored. Once ESRD patients received renal transplantation during the follow-up period, they were also censored on the date of transplantation. The date of mortality was defined as the date when an enrolled subject withdrew from NHI due to death or 1 month after stopping receiving dialysis therapy and without any subsequent medical visit. Furthermore, the subtype of dementia (Alzheimer’s disease [ICD-9: 331.0], vascular dementia [ICD-9: 290.4], or unspecified dementia [ICD-9: 290.0-290.3, 294.1, 331.1 and 331.2]) or the medical specialists of primary physicians responsible for the diagnosis of dementia were also recorded to facilitate further analyses in this study.

### Identification of comorbidities

The comorbidities listed in the Additional file 1: Table S2 were identified and considered to reduce potential confounding for risk estimation of dementia. The rationale for identifying these comorbidities as potential confounders is mainly derived from prior evidence in numerous studies [17–19]. Individuals were defined as having these comorbidities if the related diagnostic codes were identified once or more at inpatient care or twice or more at ambulatory care with a minimum interval of > 30 days within 1 year before enrollment.

### Statistical analyses

Continuous variables were compared by Student’s *t* test, and comparisons of difference between categorical variables were analyzed by the chi-square test or Fisher exact test. The Poisson assumption was used to estimate age- and sex-specific IRs, and the corresponding 95% confidence intervals (CIs) were estimated by the exact method. Since ESRD patients are at higher risk of mortality than non-ESRD patients, cumulative incidence rates (CIRs) were estimated based on the cumulative incidence competing risk analysis [30, 31]. In addition, the competing risk regression analyses with both cause-specific and subdistribution hazard models (i.e., Fine and Gray model) were conducted. The relative hazard estimated from cause-specific models may be better suited for studying the etiology of diseases, whereas that derived from subdistribution models has been used to predict an individual’s risk or allocating resources [32, 33]. A forest plot was used to reveal the hazard ratios obtained in subgroups defined on the basis of selected comorbidities, and interaction between ESRD and selected comorbidities was checked in Cox regression analyses. When constructing Cox regression models (Table 3, Fig. 3, Additional file 1: Table S4, and Additional file 1: Figures S2 and S3), multiple factors, including age, sex, diabetes mellitus, stroke, anemia, heart failure, hypertension, hyperlipidemia, coronary artery disease, peripheral vascular disease, malignancy, depression, obstructive sleep apnea, insomnia, alcoholism, traumatic brain injury, Parkinson’s disease, myocardial infarction, atrial fibrillation, hyperthyroidism, and hypothyroidism, were treated as covariates to reduce the potential confounding effect. The fulfillment of assumption of proportional subdistribution hazards in Cox regression models was checked by *log(−log(survival function))* versus log of survival time graph stratified by the covariate. The severity of multicollinearity between independent variables was evaluated by the variance inflation factors. All statistical analyses were performed using SAS, version 9.4 (SAS Institute, Cary, NC). A *P* value < 0.05 was considered as statistically significant.

## Results

### Baseline characteristics of ESRD and non-ESRD groups

A total of 99,158 ESRD patients and 927,142 non-ESRD subjects were finally enrolled for analysis (Fig. 1). Table 1 summarizes the differences in the demographic data between the ESRD and non-ESRD groups. The mean age (± standard deviation) of the ESRD group was greater than that of the non-ESRD group (61.55 ± 14.20 vs. 32.24 ± 19.67 years, respectively). Male subjects slightly dominated in the non-ESRD group, and the proportion of men and that of women were nearly identical in the ESRD group. In addition, ESRD patients were more likely to have the concomitant comorbidities than those non-ESRD individuals.

### Overall and age- and sex-specific incidence rates (IRs) of dementia in ESRD and non-ESRD groups

**Fig. 2:**
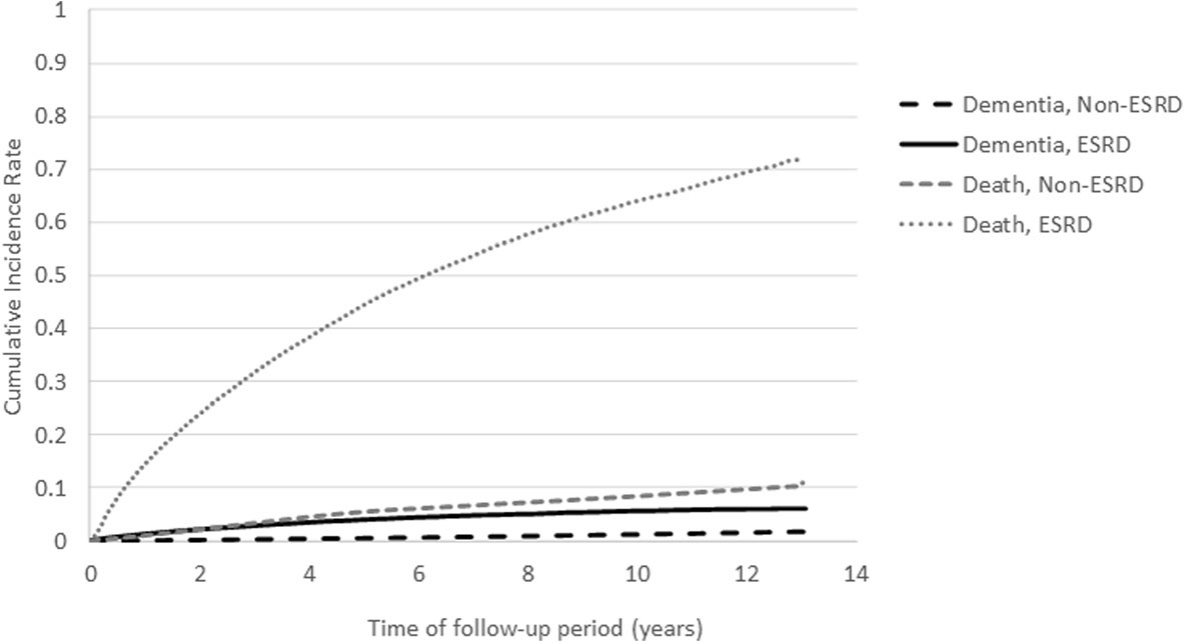
Cumulative incidence rates of dementia and all-cause mortality estimated by the cumulative incidence competing risk analysis between patients with and without end-stage renal disease (ESRD)

The overall IR of dementia was much higher in the ESRD group than in the non-ESRD group (10.73 vs. 1.40 per 1000 person-years, respectively, *P* < 0.0001) (Table 2). The difference in IRs between the ESRD and non-ESRD groups was still remarkable even after stratification by sex and age, except for those under 18 years. The IRs generally increased along with age. Nevertheless, the difference in IR ratios between the ESRD and non-ESRD groups gradually decreased while aging (from up to 3.57- to 1.30-fold in the male group and 6.78- to 1.36-fold in the female group). Moreover, individuals in the ESRD group still had a higher CIR of dementia than those in the non-ESRD group (0.061 vs. 0.017, *P* < 0.0001) throughout the study period after accounting for competing risk of mortality (Fig. 2).

**Table 2:** Overall and age- and sex-specific incidence rates of dementia between end-stage renal disease (ESRD) and non-ESRD populations

Characteristics	Non-ESRD population		ESRD population		*P* value
	No. of events	Incidence rates (per 1000 patient-years)	No. of events	Incidence rates (per 1000 patient-years)	
Male					
Age (years)					
≤ 18 years	158	0.10 (0.08–0.11)	0	0.00 (0.00–0.00)	
18–29 years	286	0.23 (0.20–0.26)	3	0.47 (0.10–1.37)	
30–39 years	313	0.30 (0.27–0.34)	14	0.96 (0.53–1.62)	
40–49 years	458	0.56 (0.51–0.61)	65	2.00 (1.55–2.55)	
50–59 years	776	1.86 (1.73–1.99)	174	3.92 (3.36–4.55)	
60–69 years	2361	7.42 (7.12–7.72)	434	10.57 (9.60–11.61)	
70–79 years	2870	17.35 (16.73–18.00)	671	23.70 (21.94–25.56)	
≥ 80 years	867	32.60 (30.47–34.85)	300	42.56 (37.88–47.65)	
Total	8089	1.43 (1.40–1.46)	1661	9.49 (9.04–9.96)	< 0.0001
Female					
Age (years)					
≤ 18 years	108	0.07 (0.06–0.09)	0	0.00 (0.00–0.00)	
18–29 years	108	0.09 (0.07–0.10)	3	0.61 (0.13–1.77)	
30–39 years	141	0.14 (0.12–0.16)	11	0.83 (0.41–1.48)	
40–49 years	374	0.45 (0.40–0.50)	54	1.53 (1.15–2.00)	
50–59 years	829	1.88 (1.75–2.01)	183	4.12 (3.55–4.77)	
60–69 years	2198	7.02 (6.73–7.32)	650	13.15 (12.16–14.20)	
70–79 years	2756	19.18 (18.47–19.91)	978	28.52 (26.76–30.36)	
≥ 80 years	1042	32.35 (30.42–34.38)	384	44.15 (39.85–48.80)	
Total	7556	1.37 (1.34–1.40)	2263	11.86 (11.37–12.36)	< 0.0001
Overall IR	15,645	1.40 (1.38–1.42)	3924	10.73 (10.39–11.07)	< 0.0001

*IR* incidence rate

### Cause-specific and subdistribution hazard ratios (HRs) and subgroup analyses stratified by selected comorbidities for risk of dementia between ESRD and non-ESRD groups

**Fig. 3:**
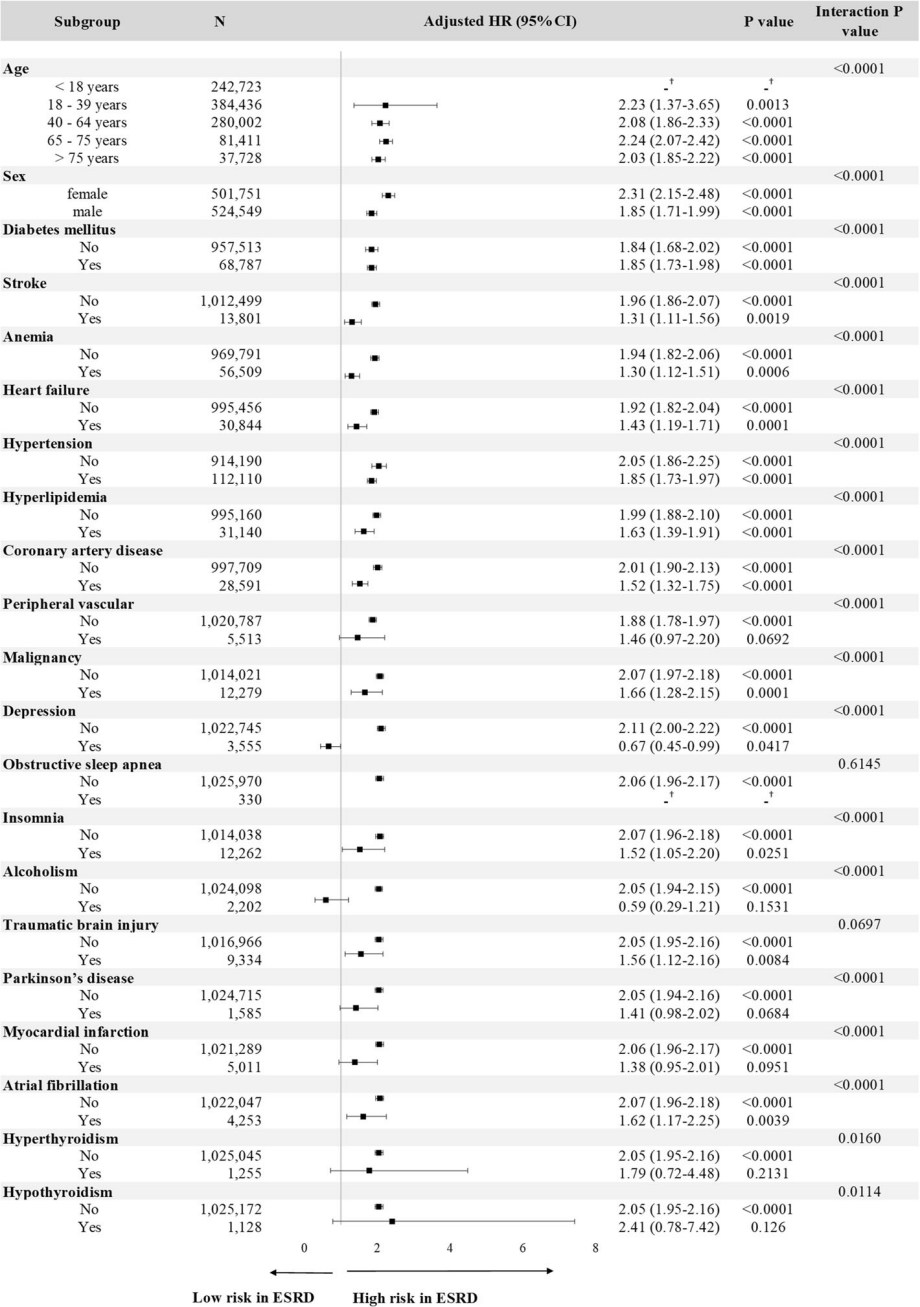
Stratified analysis of risk for dementia between the end-stage renal disease (ESRD) and non-ESRD population by using multivariable cause-specific hazard models. HR, hazard ratio; CI, confidence interval. Hazard ratios were adjusted for age, sex, and diabetes mellitus, stroke, anemia, heart failure, hypertension, hyperlipidemia, coronary artery disease, peripheral vascular disease, malignancy, depression, obstructive sleep apnea, insomnia, alcoholism, traumatic brain injury, Parkinson’s disease, myocardial infarction, atrial fibrillation, hyperthyroidism and hypothyroidism. The dagger symbol indicates that no hazard ratio (HR) was estimated because no dementia event occurred in the ESRD group

The results from the multivariable cause-specific competing risk analyses comparing the risk of dementia between ESRD and non-ESRD individuals revealed that ESRD was one of the etiological factors for developing dementia (cause-specific HR (csHR) : 2.06, 95% CI [1.95–2.17]) (Table 3). ESRD could also increase the cause-specific hazards of various subtypes of dementia, including Alzheimer’s disease (csHR : 2.71, 95% CI [2.12–3.45]), vascular dementia (csHR : 2.22, 95% CI [1.93–2.54]), or unspecified dementia (csHR : 2.01 95% CI [1.90–2.13]) (Table 3). Meanwhile, ESRD was also associated with increased cause-specific hazards for all-cause mortality (csHR ranged between 3.79 and 4.06) (Table 3), whose magnitude of relative hazard was more pronounced than that of dementia. Among the multivariable subdistribution competing risk analysis, ESRD decreased the relative incidence of dementia by 49% but it increased the relative incidence of all-cause mortality by 251% (Table 3). Similar phenomenon could also be observed in subdistribution hazards for various subtypes of dementia and their corresponding all-cause mortalities (Table 3). This suggests that the estimated individual risk of dementia in ESRD patients was strongly affected by the high mortality rate in this population. We further investigated the effect of ESRD on the cause-specific or subdistribution hazards for dementia within the subgroups stratified by various age, sex, and selected comorbidities (Fig. 3 and Additional file 1: Figure S2), and the analyses still revealed the consistent results. Among most of the age, sex, and selected comorbidity stratifications, ESRD was still suggested to be one of the etiological factors for developing dementia even after adjusting for multiple confounders (csHRs ranged between 1.30 and 2.31). When considering the individual risk of dementia over time, ESRD was associated with low risk for dementia in most of the age, sex, and selected comorbidity stratifications because of premature mortality after adjusting for multiple confounders (sdHRs ranged between 0.23 and 0.68).

**Table 3:** Estimated cause-specific hazard and subdistribution hazard ratios for dementia and mortality using multivariable regression models

Covariates	Overall		Alzheimer’s disease		Vascular dementia		Unspecified dementia	
	Dementia aHR^a^ (95% CI)	Mortality aHR^a^ (95% CI)	Dementia aHR^a^ (95% CI)	Mortality aHR^a^ (95% CI)	Dementia aHR^a^ (95% CI)	Mortality aHR^a^ (95% CI)	Dementia aHR^a^ (95% CI)	Mortality aHR^a^ (95% CI)
Cause-specific hazard models^a^								
ESRD vs. non-ESRD	2.06 (1.95–2.17)	4.06 (3.99–4.14)	2.71 (2.12–3.45)	3.79 (3.73–3.86)	2.22 (1.93–2.54)	3.82 (3.76–3.89)	2.01 (1.90–2.13)	4.00 (3.93–4.08)
Subdistribution hazard models^a^								
ESRD vs. non-ESRD	0.51 (0.49–0.54)	3.51 (3.44–3.58)	0.56 (0.45–0.69)	3.76 (3.69–3.83)	0.48 (0.42–0.53)	3.72 (3.66–3.79)	0.52 (0.49–0.55)	3.53 (3.46–3.60)

*aHR* adjusted hazard ratio, *CI* confidence interval, *ESRD* end-stage renal disease

^a^HRs were adjusted for age, sex, and selected comorbidities (diabetes mellitus, stroke, anemia, heart failure, hypertension, hyperlipidemia, coronary artery disease, peripheral vascular disease, malignancy, depression, obstructive sleep apnea, insomnia, alcoholism, traumatic brain injury, Parkinson’s disease, myocardial infarction, atrial fibrillation, hyperthyroidism, and hypothyroidism)

### Sensitivity analyses

To validate the accuracy of the diagnosis of dementia, we confined the primary physicians responsible for the diagnosis of dementia to only neurologists and psychiatrists and re-analyzed all the results by following the same study criteria (Additional file 1: Table S1, Additional file 1: Tables S3 and S4 and Additional file 1: Figures S1 and S3). The results from sensitivity analyses were robust and revealed similar results to the findings of the primary analyses. The overall and age- and sex-specific and cumulative incidence rates for dementia were higher in the ESRD group than in the non-ESRD group (Additional file 1: Table S3 and Additional file 1: Figure S1). ESRD was still associated with inverse relationships between cause-specific hazards and subdistribution hazards for either overall or any subtype of dementia (Additional file 1: Table S4). The results from the subgroup analyses were also consistent (Additional file 1: Figure S3).

## Discussion

To the best of our knowledge, this is the first study to quantify the role of ESRD in investigating the etiology and cumulative risk of dementia. To clarify this issue, we applied the competing risk regression analysis with both cause-specific and subdistribution hazard models in this population-based cohort study. Our study results clearly suggested that ESRD was indeed one of the etiological factors for overall or various subtypes of dementia because of its increased cause-specific relative hazards for dementia (csHRs 2.01–2.71) (Table 3). Since the cause-specific hazard models estimate the instantaneous rate of occurrence of the interested outcomes (dementia in our study) in individuals who are free of interested and competing events (dementia and all-cause mortality in our study), it is suggested to be better suited to address the etiology of diseases [32, 33]. Nevertheless, ESRD was associated with decreased absolute risk of dementia over time because its sdHRs were less than 1 (Table 3). Since the subdistribution hazard models estimate the instantaneous risk of failure from the interested outcome (dementia in our study) in subjects who are still free of the occurrence of the interested outcome (dementia in our study) and are attached to the cumulative incidence function, it is suggested to be better suited to predict individual risk or clinical prognosis [32, 33]. It is important to highlight that the effect of an independent variable on an outcome variable in a cause-specific model can be quite different from its effect on the corresponding outcome in the subdistribution model [34]. Only when the baseline hazard rate of the competing event is zero will the value of the csHR be equal to that of the sdHR [32]. Furthermore, the opposite direction of csHRs (> 1) and sdHRs (< 1) for the association between ESRD and risk of dementia noted in our study could happen if both the effect of cause-specific hazard ratios associated with all-cause mortality is strong enough and the baseline cause-specific hazard rate for all-cause mortality is of great magnitude [32], as shown in Table 3 and Additional file 1: Table S4. To be concrete, a stronger effect of the cause-specific hazard for all-cause mortality than for dementia in ESRD patients will result in an apparent decrease in the cumulative incidence for dementia. This is because the occurrence of the all-cause mortality will preclude the occurrence of dementia in ESRD patients and hence have a decreased cumulative risk for dementia. The effect of ESRD on the etiology and cumulative risk of dementia is still consistent after adjusting for multiple confounders among most of the age, sex, and selected comorbidity stratifications (Fig. 3 and Additional file 1: Figure S2). Therefore, we may conclude that ESRD is one of the etiological factors for developing dementia. However, ESRD patients are at lower risk for the occurrence of dementia over time because of premature mortality. Therefore, the prevention of all-cause mortality should be prior to dementia when designing the therapeutic strategies and allocating medical resources for ESRD patients.

Our study results revealed that ESRD patients had higher cumulative incidence rates than non-ESRD individuals (Fig. 2), which demonstrated the higher disease burden of dementia in the ESRD population. However, it seemed contradictory to the results that ESRD was associated with lower cumulative risk for developing dementia from the subdistribution hazard models (Table 3). Numerous risk factors of dementia, including diabetes, hypertension, hyperlipidemia, and stroke, were prevalent in the ESRD population (Table 1), and these comorbidities were also associated with increased risk for the occurrence of dementia over time (all sdHRs > 1 with statistical significance, data not shown in the table). Because the estimation of cumulative incidence rates could not adjust for the effect of these comorbidities on dementia simultaneously, this phenomenon could be interpreted that the higher cumulative incidence of dementia in the ESRD population is primarily attributable to the high prevalence of these comorbidities with risk for dementia in this population. In other words, ESRD per se does not play a crucial role, as previously believed. Therefore, our study indicated that the primary prevention strategies for dementia in the ESRD population should be weighted more on the management of these comorbidities, rather than ESRD itself. In addition, the role of ESRD for cumulative risk of dementia should be emphasized only when the mortality rates of ESRD patients can be effectively reduced. Since the subdistribution aHR of ESRD for risk of dementia is less than 1 (Table 3) and the inter-relationship between covariates in Cox models is with multiplicative effect, the effects of these risk factors for dementia might be attenuated in the ESRD population. Screening strategies and risk evaluation system should thus be reconstructed in the ESRD population to facilitate the identification or prediction of those at high risk of dementia.

Although the possible pathophysiological mechanisms of dementia related to ESRD are still not clarified, it is reported that silent brain infarction, white matter lesion, microbleeds, brain atrophy, and stroke are prevalent in ESRD patients [22, 35]. Clustering of traditional risk factors in ESRD patients, such as hypertension, diabetes mellitus, and hyperlipidemia (Table 1), can partly explain the high cerebrovascular disease burden. Non-traditional risk factors, including chronic inflammation and oxidative stress, and some specific uremic toxins, such as homocysteine and guanidine compounds, have also been suggested to contribute to not only vasculopathy-induced cognitive disorders but also neurodegenerative process [22]. Even the dialysis modality/procedure is associated with cognitive dysfunction because different time points in the dialysis cycle, the dosage or modality of the dialysis procedure reveals differential effects on cognitive function in dialysis patients [36, 37]. All of the evidence demonstrates that ESRD patients are more likely to experience higher risk for cognitive impairments and thus supports our study results that ESRD and/or hemodialysis procedure is one of the etiological factors for dementia incidence. The modifications of specific parameters of dialysis setting or the achievement of better clearance of uremic toxins with neurotoxicity should also be emphasized to prevent cognitive function decline in ESRD patients.

When individuals are associated with increased csHRs for the competing event, the value of the csHR of the interested event will be larger than that of the sdHR in these individuals because of the different modifications of risk sets of the cause-specific and subdistribution hazard models [32]. This theory could help to explain the reduced sdHRs for dementia while aging in Additional file 1: Figure S2. In the subgroup analyses of people aged between 18 and 39 years old, the estimates of csHR for dementia and mortality were 2.23 (95% CI 1.37–3.65) and 2.75 (95% CI 2.51–3.01), respectively (data not shown in the table or figure). On the other hand, the estimate of the sdHR for dementia was 1.46 (95% CI, 0.88–2.45), which was slightly less than that of the csHR for dementia. In the subgroup aged over 40 years old, the estimates of csHRs for dementia were around 2.03–2.24 but the csHRs for mortality increased to 4.34–5.37 (data not shown in the table or figure). Consequently, the corresponding sdHRs changed to 0.52–0.68. This again suggests that the risk of dementia is inversely related to increased mortality while aging. As the mortality rates of ESRD patients are higher in the USA and Europe than in Taiwan, this effect might be more pronounced in these areas [38, 39].

Our study has some following strengths. First, the data used in this study was from two representative national cohorts of the ESRD and non-ESRD populations, which could minimize potential selection bias and make the study results more generalizable. The large event numbers also allow adjustment for as many potential confounders as possible. The coverage of all nearly medical services by the NHI program also makes it possible to collect medical information comprehensively, which could reduce potential information bias when measuring the presence of comorbidities and dementia. Moreover, the follow-up period is more than one decade, which is more than enough to assess the association between morbidities and incident dementia. Second, we applied different approaches, including subgroup and sensitivity analyses, to validate the study results, and these all lead to the same conclusion and suggest the robustness of our findings. However, there are still some limitations to our study, as follows. First, while we tried our best to control for as many confounders as possible in the regression models, there are still residual confounding due to incomplete adjustment for all risk factors for dementia, such as low education level, medications, and disease severity. Failure to consider these confounders might overestimate the risk of dementia in the ESRD population. However, low education level is associated with multiple chronic illnesses, including hypertension, diabetes, and cardiovascular disease [40]. The prescriptions for medications are also closely correlated with some specific illnesses; for example, the use of statin is associated with hypercholesterolemia. The higher disease severity of specific illnesses should combine with more risk factors for developing dementia. For example, patients with poor controlled or long duration of diabetes have higher chance to have stroke, hypertension, hyperlipidemia, etc. The adjustment of these comorbidities, as many as possible, in our regression models might minimize, at least to some extent, the potential confounding. Second, the identification of dementia or comorbidities was solely dependent on ICD-9 codes, and coding errors would thus potentially lead to information bias. Nevertheless, misclassification of these illnesses should be non-differential and is likely to result in estimates of hazard ratios toward the null. In addition, various criteria for the identification of dementia were used in this study and produced essentially similar results, suggesting that the potential information bias is small and the study results are robustness. Third, the observational cohort study design could not clarify the causal inference and thus our study results should be interpreted with caution. Fourth, the non-ESRD group might have enrolled CKD-ND patients, who were also at risk for dementia, and thus might lead to underestimation of dementia risk associated with ESRD. In addition, ESRD patients have a higher frequency of medical utilizations than non-ESRD individuals, which indicate that ESRD patients have a higher chance to be detected as having dementia. To minimize these two potential biases, we performed the analysis again after excluding patients with CKD-ND in the non-ESRD population and adjusted the annual medical utilization as one of the covariates in the cause-specific and subdistribution hazard models. The results still showed similar values of cause-specific and subdistribution hazard ratios of risk of overall dementia (2.18 [95% CI 2.05–2.31] and 0.59 (95% CI 0.55–0.62), respectively) as shown in Table 3 and suggested the robustness of our study results. Fifth, we excluded the dementia cases diagnosed within the first 3 months following dialysis to avoid potential mis-ascertainment of dementia, because various medical conditions could lead to neurological complications soon after dialysis initiation. However, this way of doing might have underestimated the incidence of dementia associated with ESRD.

## Conclusions

In summary, this study demonstrated the high dementia burden in the ESRD population and showed that ESRD was one of the etiological factors for developing dementia. However, ESRD per se is not associated with increased cumulative risk of dementia over time due to high premature mortality in ESRD. The findings of our study might facilitate better decision-making in healthcare policies and may spur the development of more effective screening and therapeutic strategies for reducing the burden of dementia in the ESRD population.

## Additional file

Additional file 1: **Table S1.** Validations of the effect of end-stage renal disease on risk of dementia. Demographic and baseline characteristics of the end-stage renal disease (ESRD) and non-ESRD population in the sensitivity analyses, for which dementia is diagnosed by neurologists and psychiatrists. **Table S2.** International Classification of Disease, Ninth Edition (ICD-9-CM) codes used to identify the associated comorbidities in the study. **Table S3.** Overall and age- and sex-specific incidence rates (IRs) of dementia between end-stage renal disease (ESRD) and non-ESRD population in the sensitivity analyses, for which dementia is diagnosed by neurologists and psychiatrists. **Table S4.** Estimated cause-specific hazard and subdistribution hazard ratios for risk of dementia and all-cause mortality using multivariable Cox regression models in the sensitivity analyses, for which dementia is diagnosed by neurologists and psychiatrists. **Figure S1.** Cumulative incidence rates of dementia after accounting for competing risk of mortality between patients with and without end-stage renal disease (ESRD), for which dementia is diagnosed by neurologists and psychiatrists. **Figure S2.** Stratified analysis of risk for dementia between the end-stage renal disease (ESRD) and non-ESRD population by using multivariable subdistribution hazard models^*^.** Figure S3.** Stratified analysis of risk for dementia between the end-stage renal disease (ESRD) and non-ESRD population by using multivariable cause-specific^*^ (A) and subdistribution^*^ (B) hazard models in the sensitivity analyses, for which dementia is diagnosed by neurologists and psychiatrists (DOCX 223 kb)

## Abbreviations

CI: Confidence interval
CIR: Cumulative incidence rate
CKD-ND: Non-dialysis-dependent chronic kidney disease
csHR: Cause-specific hazard ratio
ESRD: End-stage renal disease
ICD-9: International Classification of Disease, Ninth Edition
IR: Incidence rate
LHID: Longitudinal Health Insurance Datasets
NHI: National Health Insurance
sdHR: Subdistribution HR
