# Supplementary material for: Risk of dementia in patients with end-stage renal disease under maintenance dialysis—a nationwide population-based study with consideration of competing risk of mortality
Source: Alzheimers Res Ther. 2019 Apr 9;11:31. doi: 10.1186/s13195-019-0486-z (PMC6456981; doi:10.1186/s13195-019-0486-z)
Supplement: Supplementary file 1 — Table S1. Validations of the effect of end-stage renal disease on risk of dementia. Demographic and baseline characteristics of the end-stage renal disease (ESRD) and non-ESRD population in the sensitivity analyses, for which dementia is diagnosed by neurologists and psychiatrists. Table S2. International Classification of Disease, Ninth Edition (ICD-9-CM) codes used to identify the associated comorbidities in the study. Table S3. Overall and age- and sex-specific incidence rates (IRs) of dementia between end-stage renal disease (ESRD) and non-ESRD population in the sensitivity analyses, for which dementia is diagnosed by neurologists and psychiatrists. Table S4. Estimated cause-specific hazard and subdistribution hazard ratios for risk of dementia and all-cause mortality using multivariable Cox regression models in the sensitivity analyses, for which dementia is diagnosed by neurologists and psychiatrists. Figure S1. Cumulative incidence rates of dementia after accounting for competing risk of mortality between patients with and without end-stage renal disease (ESRD), for which dementia is diagnosed by neurologists and psychiatrists. Figure S2. Stratified analysis of risk for dementia between the end-stage renal disease (ESRD) and non-ESRD population by using multivariable subdistribution hazard models*. Figure S3. Stratified analysis of risk for dementia between the end-stage renal disease (ESRD) and non-ESRD population by using multivariable cause-specific* (A) and subdistribution* (B) hazard models in the sensitivity analyses, for which dementia is diagnosed by neurologists and psychiatrists (DOCX 223 kb) [file 13195_2019_486_MOESM1_ESM.docx]

**Table S1. Demographic and baseline characteristics of the end-stage renal disease (ESRD) and non-ESRD population in the sensitivity analyses, for which dementia is diagnosed by neurologists and psychiatrists.**

|  | **Non-ESRD population** | **ESRD population** | **P value** |
| --- | --- | --- | --- |
| **Number of patients** | 927,514 | 100,492 | - |
| **Age, No (%)** |  |  |  |
| Mean (SD) | 32.25 (19.68) | 61.75 (14.25) | <0.0001 |
| ≤ 18 years | 242,490 (26.14) | 264 (0.26) | <0.0001 |
| 18-29 years | 208,898 (22.52) | 1,937 (1.93) |  |
| 30-39 years | 168,818 (18.20) | 4,809 (4.79) |  |
| 40-49 years | 132,854 (14.32) | 12,795 (12.73) |  |
| 50-59 years | 70,888 (7.64) | 21,745 (21.64) |  |
| 60-69 years | 57,402 (6.19) | 25,681 (25.56) |  |
| 70-79 years | 35,380 (3.81) | 23,883 (23.77) |  |
| ≥ 80 years | 10,784 (1.16) | 9,378 (9.33) |  |
| **Sex, No of male (%)** | 475,469 (51.26) | 49,861 (49.62) | <0.0001 |
| **Median (IQR) length of follow-up (years)** | 13.00 (0.00-0.00) | 2.88 (4.22) | <0.0001 |
| **Comorbidities, No (%)** |  |  |  |
| Diabetes mellitus | 16,873 (1.82) | 52,833 (52.57) | <0.0001 |
| Stroke | 3,242 (0.35) | 11,088 (11.03) | <0.0001 |
| Anemia | 3,982 (0.43) | 53,329 (53.07) | <0.0001 |
| Heart failure | 2,216 (0.24) | 29,228 (29.08) | <0.0001 |
| Hypertension | 35,188 (3.79) | 78,220 (77.84) | <0.0001 |
| Hyperlipidemia | 4,317 (0.47) | 27,155 (27.02) | <0.0001 |
| Coronary artery disease | 4,494 (0.48) | 24,615 (24.49) | <0.0001 |
| Peripheral vascular disease | 406 (0.04) | 5,225 (5.20) | <0.0001 |
| Malignancy | 3,922 (0.42) | 8,506 (8.46) | <0.0001 |
| Depression | 527 (0.06) | 3,156 (3.14) | <0.0001 |
| Obstructive sleep apnea | 65 (0.01) | 268 (0.27) | <0.0001 |
| Insomnia | 452 (0.05) | 12,074 (12.01) | <0.0001 |
| Alcoholism | 1,148 (0.12) | 1,066 (1.06) | <0.0001 |
| Traumatic brain injury | 7,850 (0.85) | 1,554 (1.55) | <0.0001 |
| Parkinson’s disease | 418 (0.05) | 1,317 (1.31) | <0.0001 |
| Myocardial infarction | 778 (0.08) | 4,339 (4.32) | <0.0001 |
| Atrial fibrillation | 960 (0.10) | 3,407 (3.39) | <0.0001 |
| Hyperthyroidism | 460 (0.05) | 809 (0.81) | <0.0001 |
| Hypothyroidism | 108 (0.01) | 1,054 (1.05) | <0.0001 |

SD, standard deviation.

**Table S2. *International Classification of Disease, Ninth Edition* (ICD-9-CM) codes used to identify the associated comorbidities in the study.**

| Clinical diagnosis | The associated ICD-9-CM codes |
| --- | --- |
| Diabetes | 250, 357.2, 362.0X, and 366.41. |
| Hypertension | 401-402, 405, and A codes 260 and 269. |
| Cardiovascular disease:  Congestive heart failure  Coronary artery disease  Myocardial infarction  Peripheral vascular disease | 398.91, 425, 428, 402.X1, 404.X1, and 404.X3.  414.  410.X, 412.  440–444, 447.1. |
| Stroke  Ischemic stroke  Hemorrhagic stroke | 433.xx, 434.xx, and 436.xx.  430.xx, 431.xx, and 432.xx. |
| Anemia  Hyperlipidemia | 280-285.  272.0-272.4 and A code 189. |
| Malignancy | 140-208, 230-234 and 209.0-209.3. |
| Depression | 296.2, 296.3, 296.82, 300.4, 309.0, 309.1, 311. |
| Obstructive sleep apnea | 780.51, 780.53, 780.57. |
| Insomnia | 307.4, 780.5. |
| Alcoholism | 291, 303, 305.0, 357.5, 425.5, 571.0, 571.1, 571.2, 571.3, 980.0, V11.3. |
| Traumatic brain injury | 800-804, and 850-854. |
| Parkinson’s disease | 332. |
| Atrial fibrillation | 427.3. |
| Hyperthyroidism | 242. |
| Hypothyroidism | 244. |

**Table S3. Overall and age- and sex-specific incidence rates (IRs) of dementia between end-stage renal disease (ESRD) and non-ESRD population in the sensitivity analyses, for which dementia is diagnosed by neurologists and psychiatrists.**

| **Characteristics** | **Non-ESRD population** | | **ESRD population** | | **P value** |
| --- | --- | --- | --- | --- | --- |
|  | **No. of events** | **Incidence rates (per 1,000 patient-years) and 95% CI** | **No. of events** | **Incidence rates (per 1,000 patient-years) and 95% CI** |  |
| **Male** |  |  |  |  |  |
| **Age (years)** |  |  |  |  |  |
| ≤ 18 years | 134 | 0.08 (0.07-0.10) | 0 | 0.00 (0.00-0.00) |  |
| 18-29 years | 238 | 0.19 (0.17-0.22) | 2 | 0.31 (0.04-1.12) |  |
| 30-39 years | 274 | 0.27 (0.23-0.30) | 7 | 0.48 (0.19-0.99) |  |
| 40-49 years | 368 | 0.45 (0.40-0.50) | 41 | 1.26 (0.91-1.71) |  |
| 50-59 years | 557 | 1.33 (1.22-1.44) | 119 | 2.67 (2.21-3.20) |  |
| 60-69 years | 1,652 | 5.15 (4.90-5.40) | 278 | 6.69 (5.92-7.52) |  |
| 70-79 years | 1,802 | 10.69 (10.20-11.19) | 381 | 13.03 (11.75-14.41) |  |
| ≥ 80 years | 448 | 16.23 (14.76-17.81) | 164 | 21.97 (18.73-25.60) |  |
| **Total** | 5,473 | 0.97 (0.94-0.99) | 992 | 5.60 (5.26-5.96) | <0.0001 |
| **Female** |  |  |  |  |  |
| **Age (years)** |  |  |  |  |  |
| ≤ 18 years | 84 | 0.06 (0.04-0.07) | 0 | 0.00 (0.00-0.00) |  |
| 18-29 years | 91 | 0.07 (0.06-0.09) | 2 | 0.40 (0.05-1.46) |  |
| 30-39 years | 111 | 0.11 (0.09-0.13) | 10 | 0.75 (0.36-1.38) |  |
| 40-49 years | 300 | 0.36 (0.32-0.40) | 37 | 1.05 (0.74-1.44) |  |
| 50-59 years | 666 | 1.51 (1.39-1.63) | 129 | 2.89 (2.42-3.44) |  |
| 60-69 years | 1,600 | 5.07 (4.82-5.33) | 418 | 8.33 (7.55-9.17) |  |
| 70-79 years | 1,710 | 11.61 (11.07-12.17) | 552 | 15.52 (14.25-16.87) |  |
| ≥ 80 years | 462 | 13.64 (12.43-14.94) | 181 | 19.39 (16.67-22.43) |  |
| **Total** | 5,024 | 0.91 (0.88-0.93) | 1,329 | 6.86 (6.49-7.24) | <0.0001 |
| **Overall IR** | 10,497 | 0.94 (0.92-0.96) | 2,321 | 6.26 (6.01-6.52) | <0.0001 |

SD, standard deviation; CI, confidence interval.

**Table S4. Estimated cause-specific hazard and subdistribution hazard ratios for risk of dementia and all-cause mortality using multivariable Cox regression models in the sensitivity analyses, for which dementia is diagnosed by neurologists and psychiatrists.**

| **Covariates** | **Overall** | | **Alzheimer’s disease** | | **Vascular dementia** | | **Unspecified dementia** | |
| --- | --- | --- | --- | --- | --- | --- | --- | --- |
|  | **Dementia**  **aHR**^*^ **(95% CI)** | **Mortality**  **aHR**^*^ **(95% CI)** | **Dementia**  **aHR**^*^ **(95% CI)** | **Mortality**  **aHR**^*^ **(95% CI)** | **Dementia**  **aHR**^*^ **(95% CI)** | **Mortality**  **aHR**^*^ **(95% CI)** | **Dementia**  **aHR**^*^ **(95% CI)** | **Mortality**  **aHR**^*^ **(95% CI)** |
| Cause-specific hazard models^*^ | | | | | | | | |
| ESRD vs. non-ESRD | 2.24 (2.09-2.39) | 3.98 (3.91-4.05) | 2.90 (2.20-3.84) | 3.86 (3.79-3.93) | 2.06 (1.74-2.44) | 3.88 (3.81-3.95) | 2.25 (2.09-2.42) | 3.94 (3.87-4.01) |
| Subdistribution hazard models^*^ | | | | | | | | |
| ESRD vs. non-ESRD | 0.53 (0.50-0.57) | 3.63 (3.56-3.69) | 0.56 (0.44-0.70) | 3.83 (3.77-3.9) | 0.43 (0.37-0.49) | 3.82 (3.75-3.89) | 0.56 (0.52-0.60) | 3.65 (3.59-3.72) |

^*^HRs were adjusted for age, sex, and selected comorbidities (diabetes mellitus, stroke, anemia, heart failure, hypertension, hyperlipidemia, coronary artery disease, peripheral vascular disease, malignancy, depression, obstructive sleep apnea, insomnia, alcoholism, traumatic brain injury, Parkinson’s disease, myocardial infarction, atrial fibrillation, hyperthyroidism and hypothyroidism).

aHR, adjusted hazard ratio; CI, confidence interval; ESRD, end-stage renal disease.

**Figure S1. Cumulative incidence rates of dementia after accounting for competing risk of mortality between patients with and without end-stage renal disease (ESRD), for which dementia is diagnosed by neurologists and psychiatrists.**


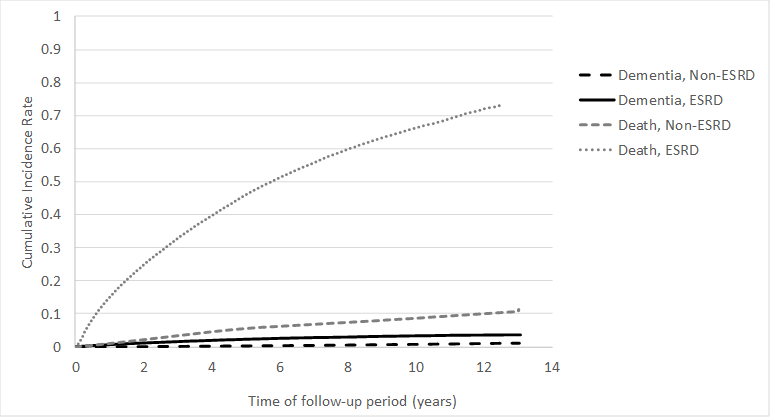


**Figure S2. Stratified analysis of risk for dementia between the end-stage renal disease (ESRD) and non-ESRD population by using multivariable subdistribution hazard models**^*^**.**

^*^ Hazard Ratios were adjusted for age, sex, and diabetes mellitus, stroke, anemia, heart failure, hypertension, hyperlipidemia, coronary artery disease, peripheral vascular disease, malignancy, depression, obstructive sleep apnea, insomnia, alcoholism, traumatic brain injury, Parkinson’s disease, myocardial infarction, atrial fibrillation, hyperthyroidism and hypothyroidism.

^†^ No Hazard ratio (HR) was estimated because no dementia event occurred in the ESRD group.

**Figure S3. Stratified analysis of risk for dementia between the end-stage renal disease (ESRD) and non-ESRD population by using multivariable cause-specific**^*^ **(Figure e-3A) and subdistribution**^*^ **(Figure e-3B) hazard models in the sensitivity analyses, for which dementia is diagnosed by neurologists and psychiatrists.**


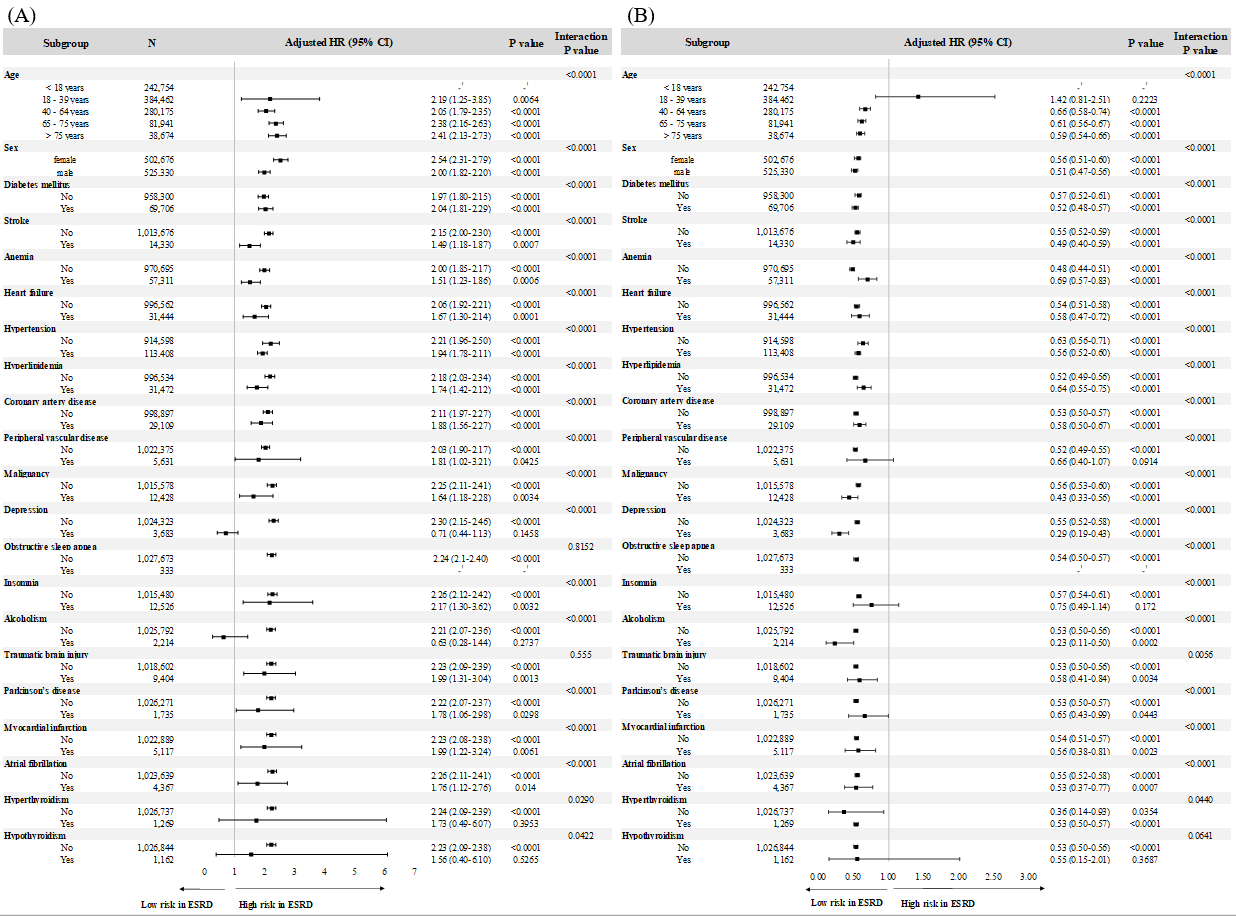


^*^ Hazard Ratios were adjusted for age, sex, and diabetes mellitus, stroke, anemia, heart failure, hypertension, hyperlipidemia, coronary artery disease, peripheral vascular disease, malignancy, depression, obstructive sleep apnea, insomnia, alcoholism, traumatic brain injury, Parkinson’s disease, myocardial infarction, atrial fibrillation, hyperthyroidism and hypothyroidism.

^†^ No Hazard ratio (HR) was estimated because no dementia event occurred in the ESRD group.
